# Comparative machine learning to predict acute kidney injury in traumatic brain injury: a MIMIC-IV cohort with SHAP interpretation

**DOI:** 10.3389/fmed.2026.1712221

**Published:** 2026-03-03

**Authors:** Zhenxing Gu, Kang Qian, Xigang Wang, Ming Li, Bo Zhang

**Affiliations:** Department of Neurosurgery, The Second Hospital of Nanjing, Nanjing, Jiangsu, China

**Keywords:** acute kidney injury, machine learning, MIMIC-IV (3.1) database, SHAP (SHapley Additive exPlanations), traumatic brain injury

## Abstract

**Background:**

AKI is a frequent and severe complication among TBI patients. Accurate early prediction is critical but remains challenging in ICU practice.

**Methods:**

We retrospectively analyzed the MIMIC-IV database. After screening 85,242 first ICU admissions and applying exclusions, 2,986 TBI patients were included. AKI was defined by KDIGO criteria. Demographic, physiological, laboratory, and intervention variables were extracted, preprocessed, and imputed. Predictors were selected using LASSO, Boruta, and logistic regression with bootstrap validation. Seven ML models (LR, DT, RF, XGBoost, LightGBM, SVM, ANN) were trained on 70% of the cohort and validated on 30%, with hyperparameters optimized by grid search and 5-fold CV. Performance was assessed by AUC, calibration, DCA, accuracy, sensitivity, specificity, PPV, NPV, and *F*_1_-score. SHAP was applied to the best-performing model (XGBoost) for global and individual interpretability.

**Results:**

Of the 2,986 TBI patients, 2,045 (68.5%) developed AKI. AKI patients were older, heavier, and had higher glucose, sodium, SBP, and temperature, with lower urine output and more frequent ventilation. Feature selection consistently retained urine output, ventilation, weight, age, glucose, sodium, SBP, and temperature as core predictors. In validation, XGBoost showed the best performance (AUC 0.775, 95% CI 0.747–0.802; accuracy 74.4%; sensitivity 88.3%; *F*_1_-score 0.83), followed by RF (AUC 0.768; sensitivity 85.9%; *F*_1_-score 0.82). LR had moderate discrimination (AUC 0.763) but poor specificity (36.5%), while LightGBM achieved the highest specificity (50.4%) but lower AUC (0.741). DT performed worst (AUC 0.728; accuracy 69.3%). Calibration and DCA supported XGBoost as having the greatest clinical benefit. SHAP analysis of XGBoost identified urine output and ventilation as dominant predictors and provided patient-level explanations consistent with observed clinical patterns.

**Conclusion:**

Ensemble ML models, particularly XGBoost, demonstrated robust predictive power, outperforming LR and DT. The XGBoost model combined high discrimination, calibration, and interpretability, offering a clinically applicable tool for early AKI risk stratification in TBI.

## Introduction

Acute kidney injury (AKI) remains a common and devastating complication in critically ill patients, contributing substantially to longer hospital stays, increased costs, and high mortality. In intensive care units (ICUs), AKI frequently arises from hemodynamic instability, sepsis, or therapeutic exposures, yet its early recognition is hindered by reliance on serum creatinine and urine output, which are delayed and often insensitive markers. As a result, clinicians are frequently unable to implement timely preventive or therapeutic measures, leaving a significant unmet need for accurate and clinically applicable early prediction tools (1–4).

Traumatic brain injury (TBI) patients represent a particularly vulnerable subgroup. They often require invasive interventions—such as mechanical ventilation, osmotic therapy, and hemodynamic support—that predispose them to kidney dysfunction. Evidence indicates that AKI in TBI is common and independently associated with poor neurological outcomes and increased mortality. Nevertheless, prediction tools specific to this population remain scarce, and conventional models lack both generalizability and interpretability. This underscores the need for predictive approaches that integrate multidimensional ICU data while providing transparency to guide individualized clinical decisions (5–8).

Machine learning (ML) offers unique advantages for AKI prediction, capturing nonlinear associations and high-dimensional interactions beyond the scope of traditional statistical models. Although previous studies in critical illness have demonstrated the promise of ML algorithms such as random forest and gradient boosting, their application in TBI-specific AKI prediction remains limited. More importantly, the clinical adoption of ML is impeded by concerns about interpretability. Emerging methods such as SHapley Additive exPlanations (SHAP) provide powerful, model-agnostic interpretability by quantifying the contribution of each feature globally and at the individual patient level, thus bridging algorithmic complexity and clinical utility. However, few studies have systematically compared multiple ML models in TBI patients using large-scale ICU datasets while also incorporating SHAP for transparency (9–14).

To address this gap, we performed a comprehensive analysis of TBI patients from the MIMIC-IV database to develop and compare seven ML models for AKI prediction. XGBoost emerged as the best-performing algorithm, achieving superior discrimination, calibration, and clinical net benefit. SHAP interpretation identified urine output, mechanical ventilation, weight, age, glucose, sodium, SBP, and temperature as dominant predictors, and provided individualized explanations for model outputs. Our findings highlight the potential of XGBoost combined with SHAP to deliver an interpretable and clinically applicable tool for early AKI risk stratification in TBI, advancing the integration of ML into neurocritical care (11, 12, 15).

## Methods

### Data source and study population

This retrospective cohort study was conducted using the MIMIC-IV (version 3.0) database. All personal data within the database were de-identified thus waiving the need for informed consent. The author (KQ) has been granted access to the dataset (Certification Number: 68550548). A total of 85,242 first ICU admissions were screened. After excluding patients with ICU stays <24 h (*n* = 18,018) and non-first ICU admissions (*n* = 9,216), 67,224 patients remained. Among them, 2,986 patients with traumatic brain injury (TBI) were identified using ICD-9/10 codes (800–804, 851, S06). Only the first ICU admission for each patient was included. The primary endpoint was acute kidney injury (AKI) during ICU stay, defined according to KDIGO criteria. Patients were stratified into AKI and non-AKI groups (1, 15).

### Data extraction and preprocessing

The model was designed to predict the risk of AKI occurring at any time during the ICU stay, using only information available within the first 24 h after ICU admission. Demographic variables, physiological measurements, laboratory indicators, and interventions (e.g., mechanical ventilation) were extracted. For time-varying predictors, Day-1 extremes were used: minimum values for urine output, and maximum values for serum creatinine, glucose, and temperature during the first 24 h. Variables with >20% missing values were removed. Outliers were truncated at the 1st and 99th percentiles. Missing data were imputed using multiple imputation by chained equations (MICE) with *m* = 5 imputed datasets. Final estimates were obtained by pooling results across all imputed datasets using Rubin’s rules. Continuous variables were expressed as mean ± SD or median (IQR), categorical variables as counts and percentages (16).

### Feature selection

Predictor selection combined least absolute shrinkage and selection operator (LASSO), Boruta, and logistic regression. LASSO regression with 10-fold cross-validation determined the *λ* values for optimal shrinkage. Boruta ranked feature importance using random forest. Stability was verified by 100 bootstrap iterations. Variables consistently retained across LASSO, Boruta, and logistic regression formed the final predictor set, which included urine output, mechanical ventilation, weight, age, glucose, sodium, SBP, and temperature (17, 18).

### Model development

Seven machine learning algorithms were developed: logistic regression (LR), decision tree (DT), random forest (RF), extreme gradient boosting (XGBoost), light gradient boosting machine (LightGBM), support vector machine (SVM), and artificial neural network (ANN). Data were split into training (70%) and validation (30%) cohorts. Hyperparameters were optimized using grid search with 5-fold cross-validation. The final configuration of XGBoost (selected as the best model) was: learning_rate = 0.01, max_depth = 5, n_estimators = 200, subsample = 0.6. To address class imbalance (AKI: 68.5% vs. non-AKI: 31.5%), we applied class weight adjustment across all applicable models. For XGBoost and LightGBM, the scale_pos_weight parameter was set to the ratio of negative to positive samples. For random forest and SVM, class_weight = “balanced” was employed to automatically adjust weights inversely proportional to class frequencies (19–21).

### Model evaluation

Model discrimination was assessed by the area under the receiver operating characteristic curve (AUC) with 95% CI. Calibration was evaluated by calibration curves. Clinical benefit was quantified using decision curve analysis (DCA). Additional performance metrics included accuracy, sensitivity, specificity, PPV, NPV, and *F*_1_-score, all reported with 95% confidence intervals calculated using 1,000 bootstrap iterations (22–25).

### Model interpretability

SHapley Additive exPlanations (SHAP) were applied to the best-performing model (XGBoost). Global interpretability was examined using summary and dependence plots, while individualized predictions were explained with force and waterfall plots (11, 12).

### External validation

To assess the generalizability of the developed models, external validation was performed using the eICU Collaborative Research Database (version 2.0), which comprises ICU data from 208 hospitals across the United States between 2014 and 2015. The same inclusion and exclusion criteria were applied: patients with TBI (identified by ICD codes), aged ≥18 years, with ICU stay ≥24 h, and excluding those with pre-existing chronic kidney disease or missing outcome data. Missing data in the eICU cohort were imputed using the same multiple imputation approach. The trained models from MIMIC-IV were directly applied to the eICU cohort without retraining, and model performance was evaluated using AUC, calibration curves, and decision curve analysis.

### Statistical analysis

Analyses were performed in R (v4.2.2) and Python (v3.9.7). Continuous variables were compared using Student’s *t*-test or Mann–Whitney *U* test; categorical variables with *χ*^2^ or Fisher’s exact test. A two-tailed *p* < 0.05 was considered statistically significant (22).

## Results

### Baseline characteristics

**Figure 1:**
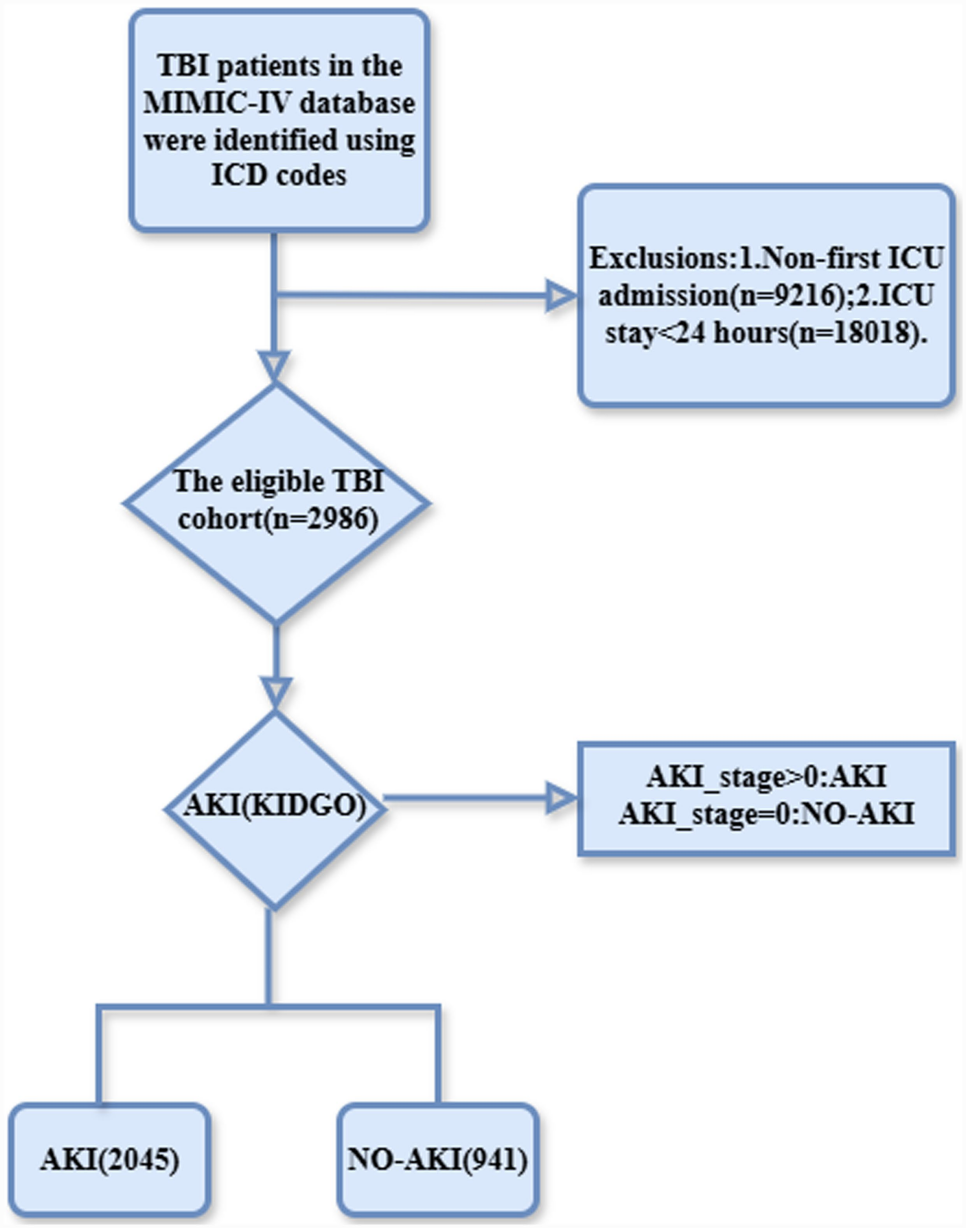
Flowchart of patient selection in the MIMIC-IV database. A total of 85,242 patients with first ICU admissions were identified. After excluding patients with ICU stays shorter than 24 h (*n* = 18,018) and non-first ICU admissions (*n* = 9,216), 67,224 patients remained eligible. Traumatic brain injury (TBI) patients were then screened using ICD codes (800–804, 851, and S06), resulting in 2,986 eligible cases. According to the Kidney Disease: Improving Global Outcomes (KDIGO) criteria, patients were stratified into acute kidney injury (AKI, *n* = 2045) and non-AKI (*n* = 941) groups.

A total of 85,242 patients with first ICU admissions were screened from the MIMIC-IV database. After excluding those with ICU stays shorter than 24 h (*n* = 18,018) and non-first ICU admissions (*n* = 9,216), 67,224 patients remained. Among them, 2,986 patients with traumatic brain injury (TBI) were identified using ICD codes. According to the KDIGO criteria, 2,045 (68.5%) patients developed acute kidney injury (AKI), while 941 (31.5%) did not (Figure 1). The raw data are provided in Supplementary Table 1. Baseline demographic and clinical variables are summarized in Table 1. Compared with the non-AKI group, patients who developed AKI were significantly older (65.0 ± 20.5 vs. 58.2 ± 22.8 years, *p* < 0.001) and had a higher body weight (79.3 ± 23.8 vs. 70.7 ± 16.7 kg, *p* < 0.001). Sex distribution differed between groups (male: 64.8% vs. 60.7%, *p* = 0.03).

**Table 1:** Baseline characteristics of the study population.

Variable	Total (*n* = 2,986)	NO-AKI (*n* = 941)	AKI (*n* = 2,045)	Statistic	*p*-value
Age (year)	62.89 ± 21.45	58.23 ± 22.82	65.03 ± 20.45	−7.81	<0.0001
Sex, *n* (%)				4.53	0.03
Female	1,090 (36.50)	370 (39.32)	720 (35.21)		
Male	1,896 (63.50)	571 (60.68)	1,325 (64.79)		
Weight (kg)	76.60 ± 22.18	70.68 ± 16.65	79.33 ± 23.82	−11.44	<0.0001
Heart_rate (hearts/min)	82.46 ± 15.65	81.49 ± 14.94	82.91 ± 15.95	−2.37	0.02
SBP (mmHg)	154.57 ± 19.78	150.92 ± 18.90	156.25 ± 19.95	−7.03	<0.0001
DBP (mmHg)	91.94 ± 19.24	91.04 ± 19.02	92.35 ± 19.32	−1.75	0.08
MBP (mmHg)	108.96 ± 23.38	107.23 ± 24.05	109.76 ± 23.02	−2.70	<0.01
Respiratory_rate (breaths/min)	26.54 ± 5.60	25.94 ± 5.36	26.81 ± 5.69	−4.05	<0.0001
Temperature (°C)	37.61 ± 0.71	37.54 ± 0.60	37.65 ± 0.75	−4.28	<0.0001
Myocardial_infarction, *n* (%)				11.03	<0.001
No	2,769 (92.73)	895 (95.11)	1,874 (91.64)		
Yes	217 (7.27)	46 (4.89)	171 (8.36)		
Congestive_heart_failure, *n* (%)				34.10	<0.0001
No	2,617 (87.64)	874 (92.88)	1,743 (85.23)		
Yes	369 (12.36)	67 (7.12)	302 (14.77)		
Chronic_pulmonary_disease, *n* (%)				6.27	0.01
No	2,620 (87.74)	847 (90.01)	1,773 (86.70)		
Yes	366 (12.26)	94 (9.99)	272 (13.30)		
Diabetes, *n* (%)				35.87	<0.0001
No	2,392 (80.11)	815 (86.61)	1,577 (77.11)		
Yes	594 (19.89)	126 (13.39)	468 (22.89)		
Malignant_cancer, *n* (%)				3.20	0.07
No	2,871 (96.15)	914 (97.13)	1,957 (95.70)		
Yes	115 (3.85)	27 (2.87)	88 (4.30)		
Hemoglobin	11.21 ± 1.74	11.50 ± 1.67	11.08 ± 1.76	6.27	<0.0001
Platelet (*10^9^/L)	194.62 ± 70.76	202.41 ± 72.15	191.03 ± 69.84	4.04	<0.0001
Rdw (%)	14.28 ± 1.81	13.90 ± 1.69	14.46 ± 1.84	−8.14	<0.0001
WBC (*10^9^/L)	10.96 ± 5.71	10.51 ± 6.37	11.17 ± 5.37	−2.77	<0.01
Anion_Gap (mEq/L)	13.86 ± 3.08	13.51 ± 2.77	14.02 ± 3.19	−4.41	<0.0001
Bicarbonate (mEq/L)	23.91 ± 3.00	24.25 ± 2.63	23.75 ± 3.14	4.59	<0.0001
BUN (mg/dL)	18.09 ± 12.81	14.73 ± 10.23	19.64 ± 13.56	−10.93	<0.0001
Total_calcium (mg/dL)	8.51 ± 0.58	8.55 ± 0.52	8.49 ± 0.61	3.04	<0.01
Creatinine (mg/dL)	1.06 ± 0.87	0.86 ± 0.41	1.14 ± 1.00	−10.94	<0.0001
Glucose (mg/dL)	137.44 ± 67.17	126.62 ± 43.94	142.42 ± 74.99	−7.21	<0.0001
Sodium (mEq/L)	140.04 ± 4.79	139.42 ± 4.58	140.32 ± 4.86	−4.93	<0.0001
Potassium (mEq/L)	4.16 ± 0.58	4.06 ± 0.56	4.20 ± 0.58	−6.38	<0.0001
INR	1.27 ± 0.38	1.22 ± 0.34	1.29 ± 0.40	−4.71	<0.0001
PT(s)	13.96 ± 4.73	13.44 ± 3.43	14.19 ± 5.20	−4.72	<0.0001
APTT(s)	30.75 ± 12.04	29.49 ± 9.37	31.33 ± 13.05	−4.38	<0.0001
Urine_output (mL)	1848.54 ± 1174.06	2175.58 ± 1242.20	1698.05 ± 1109.72	10.09	<0.0001
Charlson_comorbidity_index	3.48 ± 2.84	2.76 ± 2.71	3.81 ± 2.84	−9.73	<0.0001
SAPSII	32.67 ± 12.31	28.20 ± 11.28	34.73 ± 12.23	−14.30	<0.0001
SOFA	3.55 ± 2.56	2.82 ± 1.98	3.88 ± 2.72	−12.05	<0.0001
GCS	13.01 ± 2.74	13.14 ± 2.43	12.95 ± 2.88	1.84	0.07
Vasopressin, *n* (%)				20.18	<0.0001
No	2,893 (96.89)	932 (99.04)	1,961 (95.89)		
Yes	93 (3.11)	9 (0.96)	84 (4.11)		
Norepinephrine, *n* (%)				56.68	<0.0001
No	2,715 (90.92)	911 (96.81)	1,804 (88.22)		
Yes	271 (9.08)	30 (3.19)	241 (11.78)		
Dobutamine, *n* (%)				0.67	0.41
No	2,982 (99.87)	941 (100.00)	2,041 (99.80)		
Yes	4 (0.13)	0 (0.00)	4 (0.20)		
Milrinone, *n* (%)				0.12	0.73
No	2,980 (99.80)	940 (99.89)	2,040 (99.76)		
Yes	6 (0.20)	1 (0.11)	5 (0.24)		
CRRT, *n* (%)				10.79	<0.01
No	2,954 (98.93)	940 (99.89)	2,014 (98.48)		
Yes	32 (1.07)	1 (0.11)	31 (1.52)		
Mechanical_ventilation, *n* (%)				127.85	<0.0001
No	1,552 (51.98)	633 (67.27)	919 (44.94)		
Yes	1,434 (48.02)	308 (32.73)	1,126 (55.06)		
Neurosurgical, *n* (%)				7.64	<0.01
No	2,428 (81.31)	793 (84.27)	1,635 (79.95)		
Yes	558 (18.69)	148 (15.73)	410 (20.05)		
Los_hospital_day (day)	11.53 ± 12.60	7.65 ± 7.36	13.31 ± 14.03	−14.44	<0.0001
Los_icu_day (day)	5.14 ± 6.15	2.56 ± 2.05	6.33 ± 6.99	−22.39	<0.0001
Status_7, *n* (%)				10.41	<0.01
Alive	2,824 (94.57)	909 (96.60)	1,915 (93.64)		
Death	162 (5.43)	32 (3.40)	130 (6.36)		
Status_28, *n* (%)				29.97	<0.0001
Alive	2,764 (92.57)	908 (96.49)	1,856 (90.76)		
Death	222 (7.43)	33 (3.51)	189 (9.24)		

Demographic and clinical variables of patients with traumatic brain injury (TBI) stratified by the presence or absence of acute kidney injury (AKI) according to the KDIGO criteria. Continuous variables are expressed as mean ± standard deviation (SD) or median (interquartile range, IQR), depending on distribution. Categorical variables are summarized as counts and percentages. Comparisons between AKI and non-AKI groups were performed using the Student’s *t*-test or Mann–Whitney *U* test for continuous variables and the *χ*² test or Fisher’s exact test for categorical variables.

In terms of laboratory findings, patients with AKI exhibited significantly higher serum glucose levels (159.1 ± 60.5 vs. 148.4 ± 58.9 mg/dL, *p* < 0.001) and serum sodium concentrations (138.7 ± 6.6 vs. 137.5 ± 6.4 mmol/L, *p* < 0.001). Moreover, systolic blood pressure was slightly higher in the AKI group (131.6 ± 23.4 vs. 129.0 ± 21.9 mmHg, *p* = 0.002). Urine output was markedly reduced in the AKI group (1.07 ± 1.05 vs. 1.92 ± 1.63 L/day, *p* < 0.001). With respect to interventions, AKI patients were more likely to receive mechanical ventilation (59.1% vs. 33.2%, *p* < 0.001). Body temperature also showed a modest but significant difference (36.8 ± 0.9 vs. 36.6 ± 0.8 °C, *p* < 0.001) (Table 1). Overall, these findings indicate that older age, higher body weight, elevated glucose and sodium levels, reduced urine output, and mechanical ventilation were significantly associated with AKI development in TBI patients.

### Feature selection and importance ranking for risk prediction

**Figure 2:**
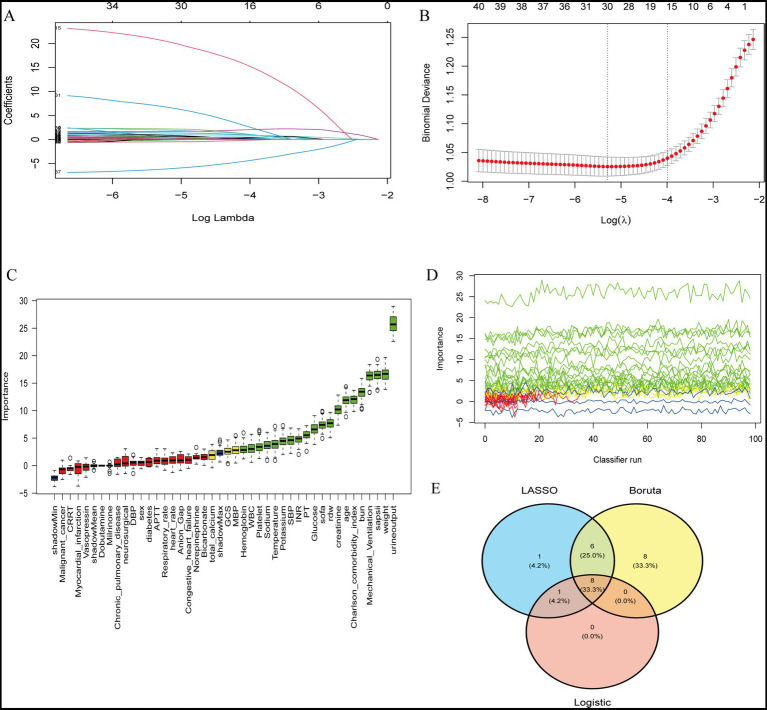
Feature selection and importance ranking for risk prediction. **(A)** LASSO coefficient profiles of candidate variables. Each curve represents the trajectory of a variable coefficient versus log(*λ*). **(B)** 10-fold cross-validation plot for LASSO regression. The dotted vertical lines indicate the optimal values of log(*λ*) corresponding to the minimum binomial deviance and the 1-SE criterion. **(C)** Variable importance ranking obtained by the Boruta algorithm. Boxplots display the distribution of importance scores for each predictor variable. **(D)** Stability of feature importance across 100 classifier runs. Green lines denote high-importance variables, yellow indicate moderate-importance, red and blue represent low-importance features. **(E)** Venn diagram showing the overlap of variables selected by LASSO, Boruta, and logistic regression models. The intersection highlights consistent predictors identified across multiple methods.

To obtain a stable and clinically coherent predictor set, we combined least absolute shrinkage and selection operator (LASSO) and Boruta with stability analysis and conventional logistic regression. LASSO coefficient profiles demonstrated progressive shrinkage of weak predictors toward zero as log(*λ*) increased (Figure 2A). 10-fold cross-validation identified both the minimum-deviance solution and the one-standard-error (1-SE) solution; we prioritized the parsimonious 1-SE model and verified robustness against the minimum-deviance choice (Figure 2B). Boruta’s random-forest-based importance ranking consistently confirmed urine output and mechanical ventilation among the top features, followed by weight, age, serum glucose, serum sodium, SBP, and temperature (Figure 2C). Across 100 classifier runs, importance profiles for high-ranked variables remained stable (Figure 2D). A Venn diagram showed substantial overlap among features retained by LASSO, Boruta, and logistic regression; their intersection constituted the final feature set used for model training and subsequent interpretation (Figure 2E) (Supplementary Tables 2, 3).

### Model performance evaluation

**Figure 3:**
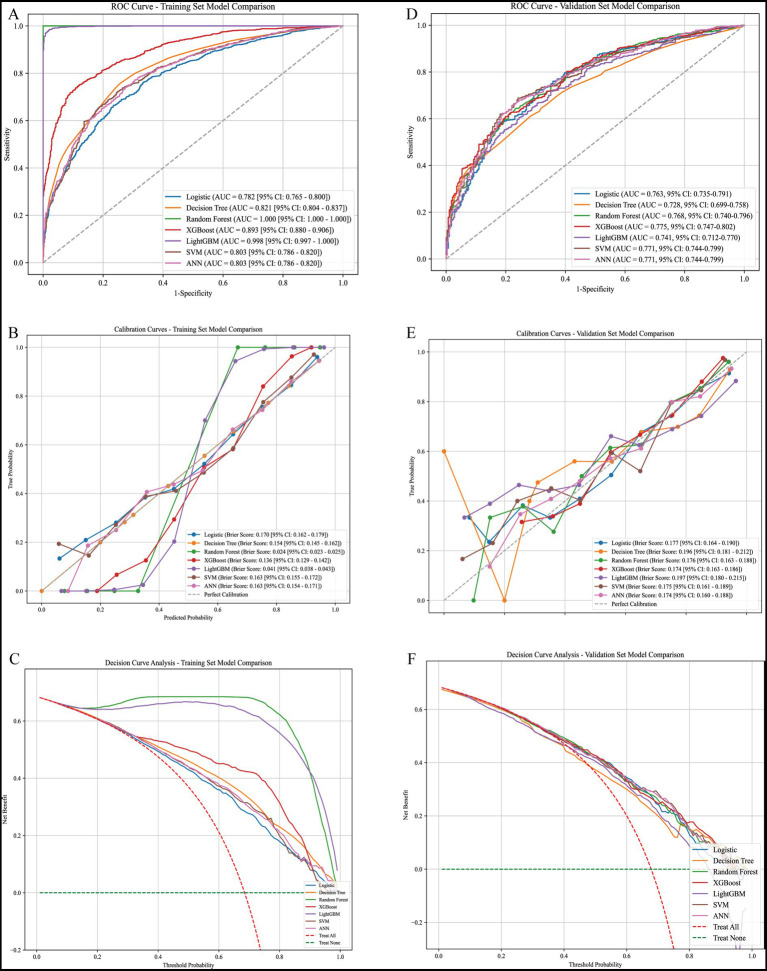
Model performance evaluation in training and validation cohorts. **(A)** Receiver operating characteristic (ROC) curves of different models in the training set. Logistic regression, decision tree, random forest, XGBoost, LightGBM, support vector machine (SVM), and artificial neural network (ANN) were compared, with corresponding AUC values indicated in the legend. **(B)** Calibration curves for each model in the training set. The closer the curve is to the diagonal, the better the agreement between predicted and observed outcomes. **(C)** Decision curve analysis (DCA) for the training set. Net benefit is plotted against threshold probability, demonstrating the clinical utility of each model. **(D)** ROC curves of model performance in the validation set. **(E)** Calibration curves for each model in the validation set. **(F)** DCA curves for the validation set, showing the clinical applicability of each predictive model.

The predictive performance of seven machine learning models was compared in both the training and validation cohorts. In the training set (70%), ensemble methods, including random forest, XGBoost, and LightGBM, achieved superior discrimination with higher AUC values compared with logistic regression and decision tree models (Figure 3A). Calibration curves demonstrated good agreement between predicted and observed probabilities across most models (Figure 3B). Decision curve analysis (DCA) indicated that XGBoost and random forest provided the highest net clinical benefit across a wide range of threshold probabilities (Figure 3C).

In the validation set (30%), model generalizability was further assessed (Table 2 and Figures 3D–F). Among all models, XGBoost achieved the best overall performance with an AUC of 0.775 (95% CI: 0.747–0.802), accuracy of 74.4%, sensitivity of 88.3%, and *F*_1_-score of 0.83, indicating both high discrimination and balanced classification ability. The random forest model also performed well (AUC = 0.768, sensitivity = 85.9%, *F*_1_-score = 0.82). Logistic regression maintained reasonable discrimination (AUC = 0.763) but exhibited lower specificity (36.5%). LightGBM showed competitive performance (AUC = 0.741) with the highest specificity (50.4%) among models (Table 2). In contrast, the decision tree model had the lowest overall accuracy (69.3%) and AUC (0.728), suggesting limited generalizability. Collectively, these results demonstrate that ensemble learning methods, particularly XGBoost and random forest, achieved the most robust and clinically applicable performance, consistently outperforming traditional logistic regression and single-tree classifiers.

**Table 2:** Performance of machine learning models in the validation set (30% of the cohort).

Validation-model	AUC	95% CI lower	95% CI upper	Accuracy	Precision	Sensitivity	Specificity	*F*_1_-score
Logistic	0.7627584371709879	0.7348891749642952	0.7906276993776806	0.7340782122905027	0.7557980900409277	0.9037520391517129	0.36524822695035464	0.8231797919762259
Decision tree	0.7284312704638274	0.6992925320827271	0.7575700088449276	0.6927374301675978	0.7674050632911392	0.7911908646003263	0.4787234042553192	0.7791164658634538
Random forest	0.768094940589821	0.7404446858109955	0.7957451953686464	0.7396648044692737	0.7818991097922848	0.8597063621533442	0.4787234042553192	0.818958818958819
XGBoost	0.7746404729675008	0.7472673324628655	0.8020136134721362	0.7441340782122905	0.7750716332378224	0.8825448613376835	0.4432624113475177	0.8253241800152555
LightGBM	0.741140536600604	0.7124447006786766	0.7698363725225313	0.7240223463687151	0.7832817337461301	0.8254486133768353	0.5035460992907801	0.8038125496425734
SVM	0.7711406522971551	0.7436181636985097	0.7986631408958005	0.735195530726257	0.7633053221288515	0.8890701468189234	0.40070921985815605	0.8214016578749057
ANN	0.7712679185033495	0.7437508130681487	0.7987850239385503	0.7374301675977654	0.7787610619469026	0.8613376835236541	0.46808510638297873	0.8179705654531371

Training-model	AUC	AUC 95% CI lower	AUC 95% CI upper	Accuracy	Precision	Sensitivity	Specificity	*F*_1_-score
Logistic	0.7824937903205297	0.7648111401649202	0.8001764404761391	0.7450980392156863	0.7696460707858428	0.895949720670391	0.417298937784522	0.8280090351726364
Decision tree	0.8205370842905706	0.8040892947145507	0.8369848738665905	0.7752271640363463	0.8168642951251647	0.8659217877094972	0.5781487101669196	0.840677966101695
Random forest	1	1	1	1	1	1	1	1
XGBoost	0.8932200049168793	0.8799828319582423	0.9064571778755162	0.8144428503108561	0.8175182481751825	0.9385474860335196	0.5447647951441578	0.8738621586475942
LightGBM	0.9984020142250405	0.9966899886292481	1.000114039820833	0.9823051171688187	0.9860627177700348	0.9881284916201117	0.9696509863429439	0.9870945238925706
SVM	0.802680546960436	0.7856225750446468	0.8197385188762252	0.7556193208990913	0.7723240685984625	0.9120111731843575	0.4157814871016692	0.8363752801793148
ANN	0.8030249404464187	0.7859782053723405	0.8200716755204969	0.7546628407460545	0.7838171710932674	0.8861731843575419	0.46889226100151743	0.831858407079646

Assessment of model generalizability in the independent validation cohort, which accounted for 30% of the dataset. Performance indicators included AUC, accuracy, sensitivity, specificity, PPV, NPV, and *F*_1_-score. Comparative results between training and validation sets demonstrate model robustness and predictive stability.

### External validation in eICU cohort

To evaluate the generalizability of the prediction models, external validation was conducted using the eICU Collaborative Research Database. After applying identical inclusion and exclusion criteria, 3,067 TBI patients from 208 US hospitals were included. Among them, 1,831 (59.7%) developed AKI during ICU stay. In the external validation cohort, XGBoost and random forest achieved the highest discrimination with an AUC of 0.620 (95% CI: 0.603–0.637), followed by SVM (AUC: 0.616, 95% CI: 0.599–0.633), LightGBM (AUC: 0.613, 95% CI: 0.596–0.630), logistic regression (AUC: 0.610, 95% CI: 0.593–0.627), ANN (AUC: 0.603, 95% CI: 0.585–0.620), and decision tree (AUC: 0.563, 95% CI: 0.546–0.581). The consistent ranking of model performance between internal and external validation cohorts supports the transportability of our findings. XGBoost demonstrated balanced performance with sensitivity of 0.604, specificity of 0.554, and *F*_1_-score of 0.634 in the eICU cohort (Table 3 and Supplementary Figure S1).

**Table 3:** Performance of machine learning models in the external validation (EICU database).

Model	AUC	95% CI	Accuracy	Sensitivity	Specificity	Precision	F_1_-score
XGBoost	0.62	0.603–0.637	0.584	0.604	0.554	0.667	0.634
Random forest	0.62	0.603–0.637	0.599	0.674	0.488	0.661	0.668
SVM	0.616	0.599–0.633	0.614	0.745	0.42	0.655	0.697
LightGBM	0.613	0.596–0.630	0.584	0.604	0.553	0.667	0.634
Logistic regression	0.61	0.593–0.627	0.586	0.607	0.555	0.669	0.636
ANN	0.603	0.585–0.620	0.588	0.609	0.556	0.67	0.638
Decision tree	0.563	0.546–0.581	0.602	0.683	0.482	0.661	0.672

Assessment of model generalizability in the independent validation cohort (EICU database). Performance indicators included AUC, accuracy, sensitivity, specificity, PPV, NPV, and *F*_1_-score.

### Model interpretability analysis

**Figure 4:**
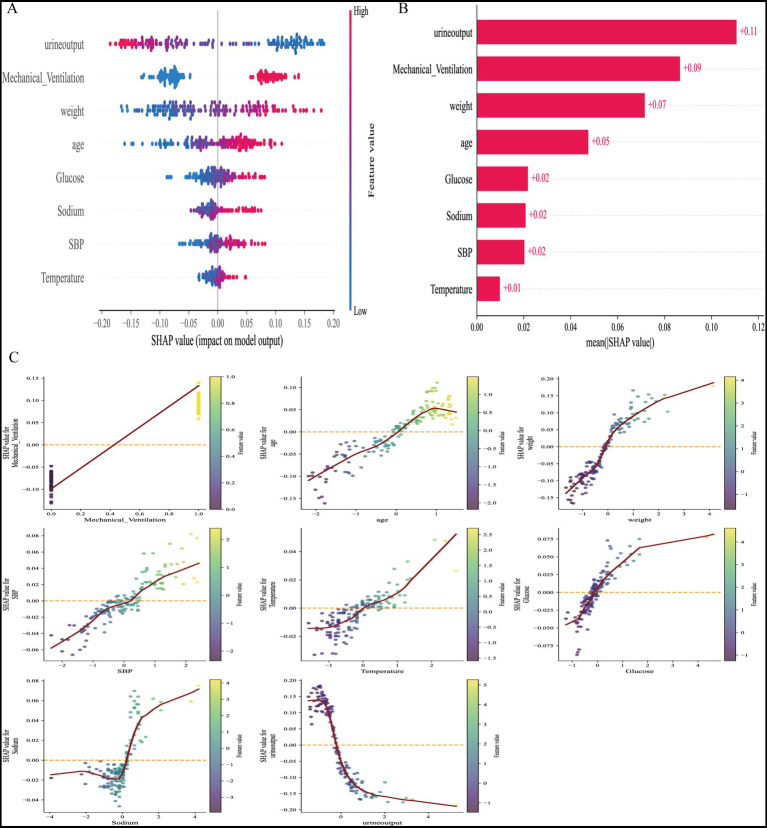
SHAP analysis of the predictive model. **(A)** SHAP summary plot showing the distribution of feature contributions. Each point represents a patient, with color indicating the feature value (red = high, blue = low). Features are ranked by importance in descending order. **(B)** Mean SHAP values of the top eight predictors. Urine output and mechanical ventilation had the largest impact on the model, followed by weight, age, glucose, sodium, systolic blood pressure (SBP), and temperature. **(C)** SHAP dependence plots for the key predictors. Each plot illustrates the relationship between feature values and SHAP contributions, with nonlinear trends reflecting their marginal effects on the model output.

To enhance clinical applicability, SHapley Additive exPlanations (SHAP) were applied to the best-performing model, XGBoost. The SHAP summary plot demonstrated that urine output and mechanical ventilation were the strongest contributors to AKI prediction, followed by weight, age, serum glucose, serum sodium, systolic blood pressure (SBP), and temperature (Figures 4A,B). These findings were consistent with the clinical associations observed in baseline characteristics. SHAP dependence plots further revealed nonlinear relationships between predictors and outcome risk (Figure 4C). For instance, decreasing urine output markedly increased the SHAP value, indicating a higher probability of AKI, while elevated glucose and sodium levels also showed a positive association with AKI risk.

**Figure 5:**
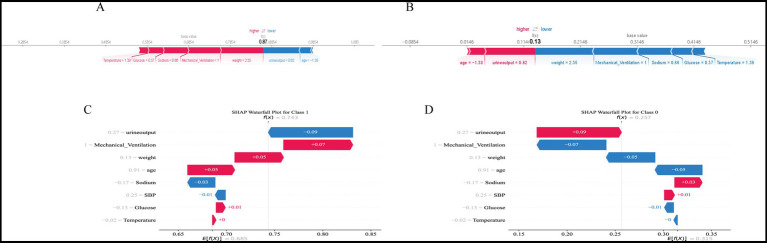
SHAP-based individualized prediction interpretation. **(A,B)** SHAP force plots for representative patients. Red segments indicate features driving the prediction towards a higher risk of outcome, while blue segments indicate protective effects. The base value represents the average model output, and the final prediction (*f*(*x*)) reflects the cumulative contribution of each feature. **(C,D)** SHAP waterfall plots for individual patients. Each bar shows the contribution of a variable to the model prediction: positive SHAP values (red) increase the predicted risk, and negative SHAP values (blue) reduce it. The plots illustrate how patient-level characteristics such as urine output, mechanical ventilation, weight, age, and biochemical indicators contribute to outcome classification.

At the individual level, SHAP-based visualization of the XGBoost model provided intuitive explanations of predictions (Figure 5). Force plots and waterfall plots illustrated how patient-specific characteristics cumulatively influenced predicted AKI risk. In high-risk patients, factors such as low urine output, need for mechanical ventilation, and higher body weight contributed strongly to increased risk, while younger age or normal sodium levels mitigated predicted probability. Conversely, in low-risk patients, protective features outweighed detrimental ones, resulting in reduced risk scores.

Overall, SHAP analysis of the XGBoost model not only confirmed the clinical relevance of the key predictors but also enabled personalized interpretation of model decisions, bridging the gap between machine learning algorithms and bedside decision-making.

## Discussion

The principal conclusion of this study is that ensemble machine learning models, particularly XGBoost, demonstrated superior ability to predict acute kidney injury in patients with traumatic brain injury compared with traditional logistic regression and single-tree methods. The combination of high discrimination, calibration, and interpretability achieved by XGBoost provides a clinically applicable tool for early AKI risk stratification in this vulnerable population (5, 6, 26, 27).

The findings of this study directly address the overarching question posed in the introduction: can advanced machine learning methods improve early AKI prediction in TBI patients while maintaining clinical interpretability? By systematically comparing seven models, I have shown that XGBoost achieved an AUC of 0.775 with balanced sensitivity and specificity, outperforming logistic regression and decision tree models. This extends prior work in the broader ICU population. For instance, Koyner et al. reported that random forest improved prediction of AKI compared with logistic regression in critically ill adults, but interpretability remained limited (39). Similarly, Tomasev et al. demonstrated that a deep learning model could predict AKI up to 48 h before onset across heterogeneous hospital populations, yet their model was criticized for limited transparency (9). My results align with these findings by confirming the superiority of ensemble learning methods, but further advance the field by providing SHAP-based interpretability specifically in the TBI population, bridging algorithmic accuracy with clinical trustworthiness (11, 12, 28).

Three specific results underscore these contributions. First, urine output and mechanical ventilation emerged as the dominant predictors of AKI across all feature selection methods and SHAP analysis. This is consistent with previous observational studies showing that oliguria is both an early warning signal and a strong predictor of AKI in ICU cohorts. The reinforcement of urine output as the most influential factor by SHAP not only validates prior evidence but also demonstrates that interpretable ML can replicate and quantify known pathophysiological associations. Similarly, the association between mechanical ventilation and AKI has been recognized as part of the “lung–kidney cross talk,” where positive pressure ventilation alters renal hemodynamics and inflammatory cascades. My analysis confirms and quantifies this relationship, highlighting ventilation as a critical marker in risk stratification for AKI in neurocritical care patients (8, 29, 30).

Second, ensemble models consistently outperformed logistic regression in discrimination, calibration, and net benefit. Logistic regression yielded an AUC of 0.763, but its specificity was only 36.5%, limiting clinical utility. By contrast, XGBoost and random forest balanced sensitivity (88.3 and 85.9%, respectively) with clinically relevant specificity, which was further validated by decision curve analysis. These findings align with the growing recognition that linear models are insufficient to capture nonlinear interactions in high-dimensional ICU data. My results extend this observation to the TBI setting, underscoring that nonlinear relationships—such as the combined effect of age, weight, and glucose dysregulation—must be modeled to achieve accurate prediction (13, 31, 32).

Third, the application of SHAP to XGBoost provided individualized explanations that mirrored observed clinical profiles. For example, low urine output, requirement for ventilation, and elevated sodium collectively drove predictions toward higher AKI risk, while younger age or normal sodium reduced risk. This patient-level transparency represents a critical advance beyond prior “black-box” models. Lundberg and Lee introduced SHAP as a theoretically grounded method for feature attribution, but its integration into clinical AKI prediction has been rare. My study translates this interpretability framework into neurocritical care, showing that transparent machine learning can be both accurate and clinically meaningful in a high-stakes context (11, 12, 28).

XGBoost was selected as the optimal model based on a comprehensive evaluation incorporating discrimination (AUC), calibration, clinical utility (DCA), and balanced classification metrics (*F*_1_-score). While SVM and ANN demonstrated comparable performance on certain individual metrics, XGBoost consistently achieved the best balance across all evaluation dimensions. We acknowledge the notable gap between training AUC (approaching 1.0) and validation AUC (0.775), which suggests some degree of overfitting despite regularization. Future work will explore additional regularization techniques and larger external datasets to further mitigate this issue.

The relatively high AKI incidence (68.5%) observed in our TBI cohort warrants discussion. This rate exceeds some previous reports (30–50%) but is consistent with studies focusing on severe TBI requiring ICU admission. The MIMIC-IV database captures patients from a tertiary academic medical center with high disease severity, which may explain the elevated AKI rate.

The predictive roles of weight, sodium, and temperature merit pathophysiological elaboration in the TBI context. Higher body weight may reflect greater osmotic load from mannitol therapy and increased contrast exposure during imaging, both nephrotoxic. Dysnatremia, particularly hypernatremia from osmotic therapy or diabetes insipidus common in TBI, directly impairs renal concentrating ability and tubular function. Elevated temperature may indicate systemic inflammation or infection, both of which accelerate kidney injury through hemodynamic compromise and inflammatory cascades.

Ethical considerations in machine learning-based clinical prediction deserve attention. Algorithmic bias may arise from underrepresentation of certain demographic groups in the training data, potentially leading to disparate model performance across populations. The MIMIC-IV database, derived from a single US academic center, may not adequately represent diverse patient populations. Future work should assess model fairness across demographic subgroups.

Nevertheless, this work also reveals areas of partial disagreement with the literature. For instance, while LightGBM achieved the highest specificity (50.4%), its AUC was lower (0.741) compared to XGBoost. This contrasts with recent studies where LightGBM often outperformed XGBoost in healthcare prediction tasks due to its efficiency and leaf-wise tree growth. The discrepancy may reflect differences in dataset characteristics: the MIMIC-IV TBI cohort is relatively small and imbalanced compared with general ICU populations, favoring the robustness of XGBoost’s depth-wise splitting strategy. Another point of divergence concerns artificial neural networks. Although ANN has demonstrated state-of-the-art performance in large-scale prediction tasks, my results showed inferior discrimination (AUC not exceeding 0.75) compared with ensemble methods. This is likely attributable to the moderate sample size and heterogeneous feature set, which may be insufficient to exploit the full capacity of neural networks without overfitting. These differences underscore the need to contextualize algorithmic performance within population-specific and dataset-specific parameters (33, 34).

The external validation using the eICU database provides important evidence for model generalizability. While the AUC values in the external cohort (0.620 for XGBoost) were lower than those in the internal validation cohort (0.775), this performance degradation is commonly observed when prediction models are transported across different healthcare settings. The eICU database encompasses 208 diverse hospitals with varying patient populations, clinical practices, and documentation standards, which may explain the performance gap. Importantly, the relative ranking of models remained consistent, with XGBoost and Random Forest maintaining their positions as top performers. This supports the robustness of our model selection and suggests that the identified predictors retain their prognostic value across different TBI populations.

First, although we performed external validation using the eICU database (*n* = 3,067 TBI patients from 208 US hospitals), with XGBoost achieving an AUC of 0.620 (95% CI: 0.603–0.637), the model performance was lower than in internal validation (AUC: 0.775). This performance gap may reflect heterogeneity in patient populations, clinical practices, and data quality across institutions. Future prospective multi-center studies with standardized data collection are warranted to further validate and refine the model before clinical implementation.

Looking forward, several avenues for extension are evident. First, future studies should validate and refine the XGBoost–SHAP model using external, prospective, multi-center datasets to confirm robustness across different healthcare systems and patient populations. Second, incorporation of longitudinal features and temporal modeling approaches such as recurrent neural networks or transformer architectures could enhance predictive accuracy while preserving interpretability through hybrid SHAP extensions. Third, integration of novel biomarkers—such as NGAL, TIMP-2, and IGFBP7—into ML frameworks may improve sensitivity for early injury detection. Fourth, embedding these models into real-time electronic health record systems with user-friendly SHAP visualizations could enable proactive clinical decision support, allowing individualized interventions to prevent AKI progression. Ultimately, combining the predictive accuracy of ensemble machine learning with transparent interpretability tools has the potential to transform AKI risk stratification and management in neurocritical care, improving outcomes for TBI patients (35–38).

## Conclusion

Seven machine learning models (LR, DT, RF, XGBoost, LightGBM, SVM, ANN) were constructed and systematically compared, identifying XGBoost as the optimal approach with balanced discrimination, calibration, and interpretability. The application of SHAP revealed urine output, ventilation, weight, age, glucose, sodium, SBP, and temperature as dominant contributors closely linked with AKI in TBI patients. These indicators not only strengthened the predictive model but also demonstrated clinical relevance for early identification and risk stratification. By incorporating these predictors, accurate recognition of vulnerable patients may be achieved, offering a practical tool to guide timely intervention. Furthermore, this framework provides meaningful support for individualized decision-making and holds promise for integration into clinical workflows after validation in prospective, multi-center studies.

## Data Availability

The original contributions presented in the study are included in the article/Supplementary material, further inquiries can be directed to the corresponding authors.

## Supplementary material

SUPPLEMENTARY FIGURE S1 Model performance evaluation in external validation (EICU database). **(A)** Receiver operating characteristic (ROC) curves of different models in external validation (EICU database). Logistic regression, decision tree, random forest, XGBoost, LightGBM, support vector machine (SVM), and artificial neural network (ANN) were compared, with corresponding AUC values indicated in the legend. **(B)** Decision curve analysis (DCA) for the external validation (EICU database). Net benefit is plotted against threshold probability, demonstrating the clinical utility of each model. **(C)** Calibration curves for each model in the external validation (EICU database). The closer the curve is to the diagonal, the better the agreement between predicted and observed outcomes.

## Glossary

AKI: Acute kidney injury
ANN: Artificial neural network
AUC: Area under the receiver operating characteristic curve
CV: Cross-validation
DCA: Decision curve analysis
DT: Decision tree
ICU: Intensive care unit
KDIGO: Kidney Disease: Improving Global Outcomes
LASSO: Least absolute shrinkage and selection operator
LightGBM: Light gradient boosting machine
LR: Logistic regression
ML: Machine learning
MIMIC-IV: Medical Information Mart for Intensive Care IV
NPV: Negative predictive value
PPV: Positive predictive value
RF: Random forest
SBP: Systolic blood pressure
SHAP: SHapley Additive exPlanations
SVM: Support vector machine
TBI: Traumatic brain injury
XGBoost: Extreme gradient boosting
