# Supplementary figures and images for: Comparative machine learning to predict acute kidney injury in traumatic brain injury: a MIMIC-IV cohort with SHAP interpretation
Source: Front Med (Lausanne). 2026 Mar 3;13:1712221. doi: 10.3389/fmed.2026.1712221 (PMC12992226; doi:10.3389/fmed.2026.1712221)

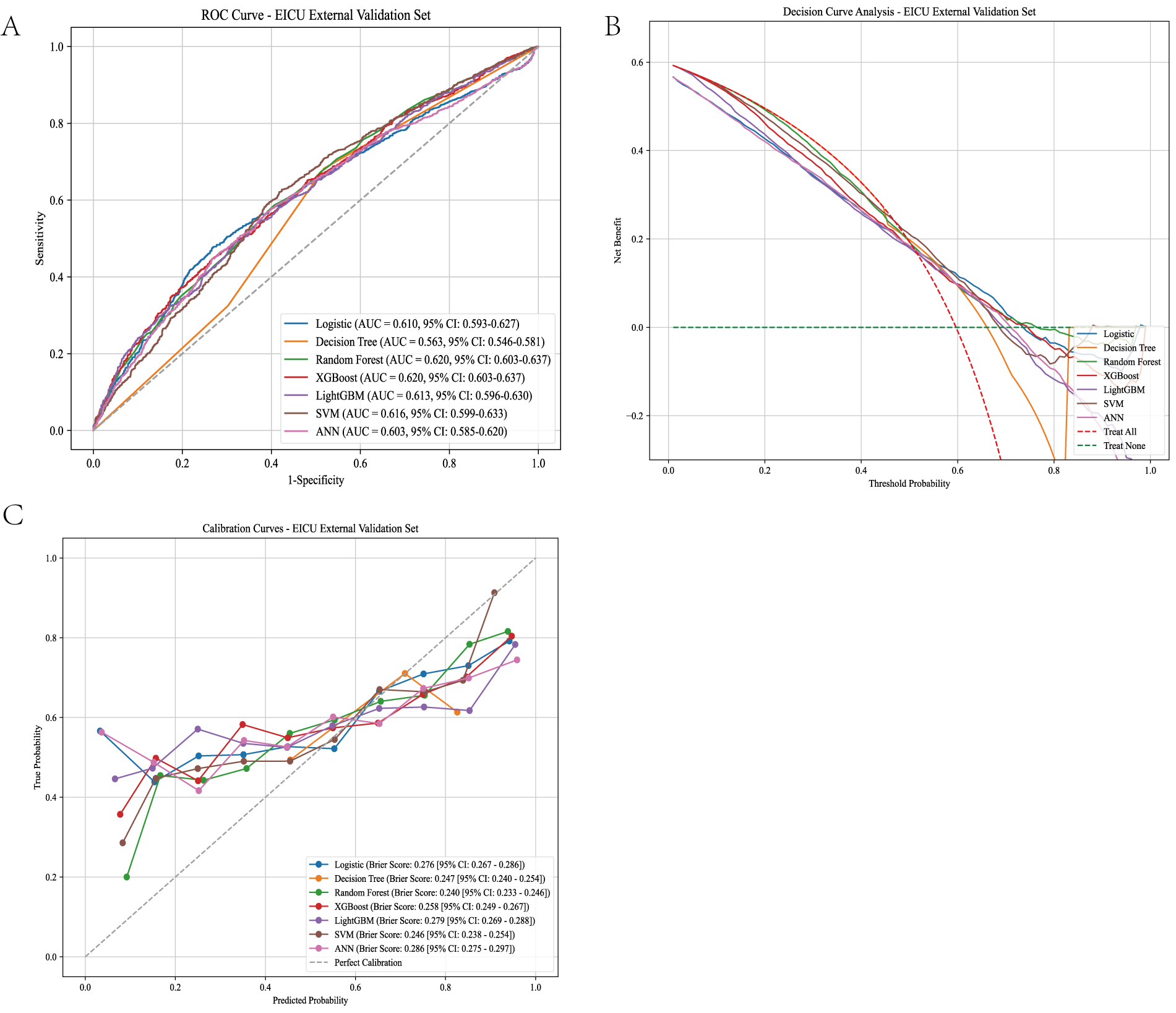

Supplement: SUPPLEMENTARY FIGURE S1 — Model performance evaluation in external validation (EICU database). (A) Receiver operating characteristic (ROC) curves of different models in external validation (EICU database). Logistic regression, decision tree, random forest, XGBoost, LightGBM, support vector machine (SVM), and artificial neural network (ANN) were compared, with corresponding AUC values indicated in the legend. (B) Decision curve analysis (DCA) for the external validation (EICU database). Net benefit is plotted against threshold probability, demonstrating the clinical utility of each model. (C) Calibration curves for each model in the external validation (EICU database). The closer the curve is to the diagonal, the better the agreement between predicted and observed outcomes. [file Image_1.tif]
